# Attentional Bias in Human Category Learning: The Case of Deep Learning

**DOI:** 10.3389/fpsyg.2018.00374

**Published:** 2018-04-13

**Authors:** Catherine Hanson, Leyla Roskan Caglar, Stephen José Hanson

**Affiliations:** ^1^Rutgers Brain Imaging Center, Newark, NJ, United States; ^2^RUBIC and Psychology Department and Center for Molecular and Behavioral Neuroscience, Rutgers University–Newark, Newark, NJ, United States

**Keywords:** attentional bias, categorization, condensation, deep learning, filtration, learning theory, neural networks

## Abstract

Category learning performance is influenced by both the nature of the category's structure and the way category features are processed during learning. Shepard (1964, 1987) showed that stimuli can have structures with features that are statistically uncorrelated (separable) or statistically correlated (integral) within categories. Humans find it much easier to learn categories having separable features, especially when attention to only a subset of relevant features is required, and harder to learn categories having integral features, which require consideration of all of the available features and integration of all the relevant category features satisfying the category rule (Garner, 1974). In contrast to humans, a single hidden layer backpropagation (BP) neural network has been shown to learn both separable and integral categories equally easily, independent of the category rule (Kruschke, 1993). This “failure” to replicate human category performance appeared to be strong evidence that connectionist networks were incapable of modeling human attentional bias. We tested the presumed limitations of attentional bias in networks in two ways: (1) by having networks learn categories with exemplars that have high feature complexity in contrast to the low dimensional stimuli previously used, and (2) by investigating whether a Deep Learning (DL) network, which has demonstrated humanlike performance in many different kinds of tasks (language translation, autonomous driving, etc.), would display human-like attentional bias during category learning. We were able to show a number of interesting results. First, we replicated the failure of BP to differentially process integral and separable category structures when low dimensional stimuli are used (Garner, 1974; Kruschke, 1993). Second, we show that using the same low dimensional stimuli, Deep Learning (DL), unlike BP but similar to humans, learns separable category structures more quickly than integral category structures. Third, we show that even BP can exhibit human like learning differences between integral and separable category structures when high dimensional stimuli (face exemplars) are used. We conclude, after visualizing the hidden unit representations, that DL appears to extend initial learning due to feature development thereby reducing destructive feature competition by incrementally refining feature detectors throughout later layers until a tipping point (in terms of error) is reached resulting in rapid asymptotic learning.

## Introduction

Categorization is a fundamental cognitive process that imposes order on an otherwise overwhelming perceptual experience through an attentional bias toward stimulus features. The ease with which stimuli are categorized is determined by the complexity of the category structure and how it interacts with attentional bias (Shepard et al., 1961). Shepard (1964, 1987) has shown that stimulus structures can be broadly classified into those in which features are independent or weakly correlated (separable structure) and those in which feature dimensions are functionally dependent or highly correlated (integral structure). He further argued that the inter-stimulus distance between exemplars within a category varies as a function of the distance metric. Specifically Shepard (1987), showed through MDS, that separable structures are judged to be more similar using an L1 metric (meaning distance is measured on each dimension separately) while integral structures are judged more similar using an L2 metric (meaning distance on each dimension measured jointly). This allows us to vary stimulus structure (integral, separable) independently of category rule (filtration, condensation) To the extent that the learner need attend to only a subset of features (defined as a “filtration rule”), the category learning is less complex than requiring an integral feature structure where many more features must be attended and integrated (defined as a “condensation rule”). This type of rule is fundamentally conjunctive, in that you **must** attend to both features to correctly assign exemplars to categories.

Not surprisingly, humans find it much easier to learn a category that has a separable feature structure with a filtration rule than one which has an integral feature structure with a condensation rule (Posner, 1964; Garner, 1974; Kemler and Smith, 1978; Kruschke, 1993) although filtration rules always show an advantage over condensation rules. The other two possible structures are even more difficult to learn due to the mismatch between the stimulus feature structure with the category rule (separable/condensation and integral/filtration). Different pairings of stimulus feature structure (separable, integral) and category rule (filtration, condensation) produce different learning responses and allow testing of attentional bias. In the present study, we paired a separable structure with a filtration rule, and an integral structure with a condensation rule to test the effect of attentional bias in networks during category learning. Inasmuch as these pairings constitute the largest expected difference in learning rate, they provide the most diagnostic cases (Gottwald and Garner, 1975; Kruschke, 1993), the other two cases mentioned before would tend to have similar learning rates, and generally fall between the two other cases.

Interestingly, when single hidden layer backpropagation (BP) neural networks (e.g., Rumelhart et al., 1986) were first used in categorization studies, the BP network was found to learn categories with an integral feature structure with a condensation rule as easily as it did categories having a separable feature structure with a filtration rule (this was independent of the different input encodings that were tried, but see footnote 1 for more details; Hanson and Gluck, 1991; Kruschke, 1993; Kurtz, 2007). That is, the BP network failed to categorize in the way humans do. This result shed doubt on the usefulness of the BP neural network and neural networks more generally as an adequate model for human attentional bias. This result consequently caused researchers to turn to various modifications of BP networks in the form of pre-defined attentional bias.

It is possible to make BP category learning more human-like by building in attentional bias (Kruschke, 1993) or increasing feature competition (Hanson and Gluck, 1991). For example, Kruschke (1993) introduced a BP network called ALCOVE that had adaptable attention strengths (weights) on the input dimensions. This network could reproduce the human like differential learning speed of the filtration/separable-condensation/integral outcome, but could do so only by having the explicit attention strengths built in. More recently in the spirit of ALCOVE, Love et al. (2004) developed the SUSTAIN model (Supervised and Unsupervised STratified Adaptive Incremental Network) which had a built-in selective attention mechanism based on receptive field tuning. Finally, Kurtz (2007, 2015) proposed a BP model called DIVergent Autoencoder (DIVA), which performed task-driven recoding of inputs into a distributed representational space as a means of solving N-way classification tasks. Another modification of BPs by Hanson and Gluck (1991) replaced the standard radial feature space (Gaussian) with a heavy tailed density (Cauchy) hidden unit kernel, producing a “greedy spotlight” effect that forced more local competition between hidden units. In effect, this approach extended the configural cue model by Gluck and Bower (1988), which implemented an implicit hypothesis search through potential (the power-set) feature combinations based feature competition between all possible features sets. To be precise, the implicit hypothesis testing was in the sense of the dynamics of learning and the incremental structure the network was constructing as new data samples updated weights. In a similar vein, the Hanson and Gluck model induced a hypothesis search by using a hidden unit receptive field that was less compact and more global than that used in the Kruschke ALCOVE model. This tactic in turn made the acquisition of category rules slower, decreasing the rate of learning, especially in the integral stimulus/condensation rule condition where more features (and more feature competition occurred) were required to learn the category rule.

Although the various models just described contributed significantly to the modeling of human category learning, all were dependent on built-in specifications to achieve human-like classification. BP models alone cannot differentially learn or distribute attention without adding some sort of designed perceptual bias (e.g., feature weights, localized kernels), which are not directly related to the learning rule itself. In effect, these models augmented the BP network's ability to learn, but did not fundamentally change the *way* it learned.

The recent advent of deep learning (DL) neural networks (Hinton et al., 2006; LeCun et al., 2015) has been revolutionary in furthering the application of artificial intelligence to a wide variety of contexts that include language translation, game playing, and self-driving vehicles. The unparalleled success of DL neural networks begs the question of how, in the present context, “human-like” DL categorization might be.

The answer to this question may lie in the concept of “feature binding” (Treisman, 1998). Feature binding refers to the way features are selected and integrated to create a meaningful unit. Thus, an attentional bias toward processing a subset of relevant features, or one biased toward integrating all relevant features, produces distinct ways of feature binding during category learning. The architecture of the basic single hidden layer BP network involves a single set of linear projections that are sensitive to stimulus covariation (and modulated through a non-linear activation function), but may not necessarily encourage feature binding. In contrast, the DL neural network architecture promotes progressive refinement of feature detectors through compression and recursion so that at each layer the feature detectors are more complex than those at earlier layers (Hinton et al., 2006; LeCun et al., 2015). It is possible that this successive processing of stimuli in DL hidden layers impose constraints that are more consistent with human attentional biases during categorization.

If network architecture is the limiting factor in binding and hypothesis testing, then an unmodified BP network would never be able to reproduce human categorization performance under any conditions as it is restricted to using a parallel, combinatorial code. Alternatively, attentional bias toward a subset of relevant features, rather than toward the integration of all available features, may depend on the complexity of the category structure itself. Specifically, less complex category structures involving separable features may encourage attention to a subset of relevant features during category learning, whereas category structures involving integral features may be inherently too complex to accommodate any attentional bias other than one in which all available features are considered. In this case, the unmodified BP network may perform more like humans during categorization.

To explore these possibilities, we compared the performance of BP and DL networks to that of humans learning categories that had either a separable or an integral feature structure. In one condition we used the Kruschke stimuli (1993) with which unmodified BP networks fail to show learning differences, and in a second condition we used computer generated realistic human faces. We then inspected the internal representation of the hidden units of the BP and DL networks to determine how input was abstracted during learning and to characterize the nature of the feature binding.

## Methods

Two types of stimuli were used in the evaluation of category learning by humans, a BP network, and a DL network. One set of stimuli were based on those used by Kruschke (1993; originally defined by Garner, 1974) in a study that illustrated the failure of a BP network to replicate the learning performance of humans. The other type consisted of computer generated realistic faces. These two stimulus sets were selected to compare low dimensionality (Kruschke) and high dimensionality (faces) stimuli.

For both kinds of stimuli, categories having a separable feature structure and categories having an integral feature structure were created through category rule assignment. For example, the category “red objects” is defined by the rule “all members must be red” and other features (e.g., size, texture, etc.) are irrelevant. Alternatively, the category “dangerous objects” is considerably more complex inasmuch as multiple features (e.g., sharpness, size, weight, etc.) must be taken into consideration. A small sharp pin is less dangerous than a large, sharp hunting knife. In this way the category rule determines where attention should be allocated most effectively during learning.

Following this logic, we were able to create low dimensional stimuli (separable) defined by a category rule involving a subset of features (filtration) and high dimensional stimuli (integral) defined by a category rule requiring the integration of all relevant features (condensation). We used these two extremes (filtration-separable, FS) and (condensation-integral, CI) in the current study inasmuch as performance on the other two possible conditions (filtration-integral, FI) and (condensation-separable, CS) are known to fall between the FS and CI conditions, and consequently provides similar diagnostic information on attentional bias.

### Stimuli

#### Low dimensional

**Figure 1:**
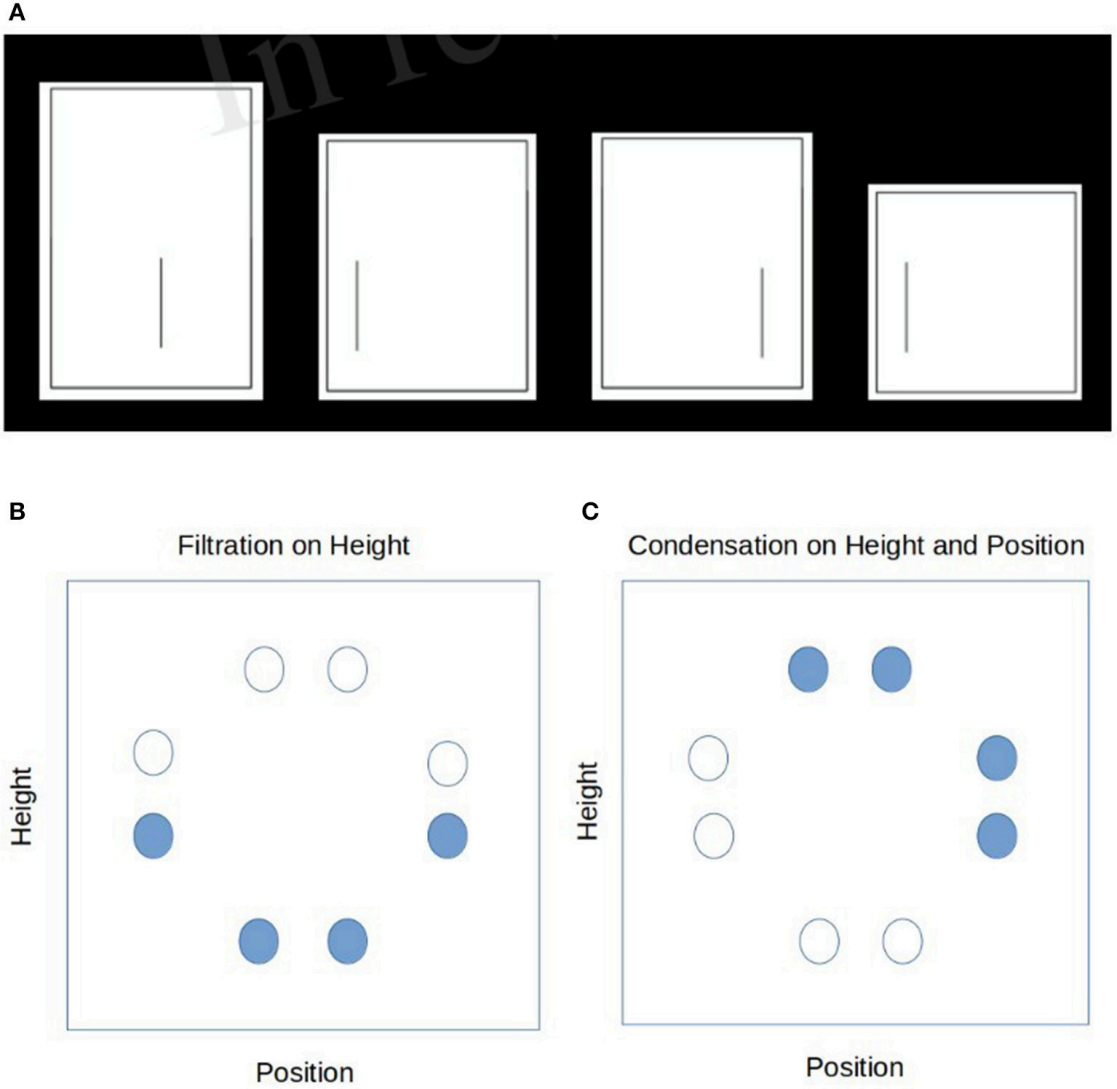
Renditions of the original stimuli from Kruschke (1993) **(A)** a sample of 4 stimuli in the low dimensional categorization **(B)** the filtration rule applied to the 8 stimuli as indicated in the 2-d feature space. **(C)** The condensation rule applied to the 8 stimuli as indicated in the 2-d feature space.

These stimuli were adopted from Kruschke's (1993) study and consisted of eight rectangles with an internal segmenting line. Two features were relevant to the category structure: (1) the height of the rectangle (four heights were used), and (2) the position of the line segment within the rectangle (four positions) (see Figure 1). Kruschke provided some evidence that the stimulus set he constructed could possess both separable or integral structure. Following Shepard (1987), he measured a separable set (say line length) with both L1 (dimensions taken independently) and L2 metrics (dimensions taken jointly), in this case the inter-stimulus similarity was higher with L1 compared to L2. When the same stimulus are grouped with a condensation rule, the inter-stimulus similarity was higher with L2 than to L1. In this way Kruschke could impose one of the category tasks and at the same time induce a separable or integral feature structure as we describe in more detail next. This allowed the manipulation of attentional bias toward a single relevant feature (the position of the line segment or the height of the rectangle) in the filtration task, or on the conjunction of the two features (*line segment position* and *rectangle length)* in the condensation task, resulting in eight distinct stimuli to be learned. Visually, these stimuli were chosen to be a symmetric circular structure in the 2D length-position feature space to be classified with either a vertical (or horizontal) separating line for filtration type rules or a diagonal separating line for condensation type rules.

#### High dimensional

Realistic, computer generated human faces, which are naturally high in dimensionality, were used. Two distinct prototypes were randomly generated using the same parameter settings (i.e., race, gender, age, etc.) in the 3D face-modeling software FaceGen Modeler Core 3.14 by Singular Inversions Inc. Each prototype was chosen to be within a standard deviation of one another (based on 12 generating parameters—gender, age, skin-tone, etc.) ensuring that there would be exemplar overlap and potential for correlation within a category set depending on included exemplar faces. In effect, the goal was to generate families of faces from each prototype that could have overlapping features, so that they could be correlated within categories and between categories similar to Kruschke stimulus construction. Moderate correlations of exemplars within a category (integral structure) could be simultaneously created with moderate between correlation (condensation rule). While high correlation of exemplars within a category (separable structure) could be created with a low correlation between categories. We describe the procedure in more detail next.

We generated 20 random samples around each prototype with the “randomness” function in the software set to 0.5, which controls for how different the random variations will be from the selected prototype. Each one of the resulting 40 faces separate feature vectors had shared “family resemblance” with either Prototype1 or Prototype2. From the 40 faces 10 were randomly selected from each prototype set, creating 20 unique face stimuli to be sorted by each category rule (C or F). The original prototypes were excluded from any further analysis or stimulus use.

Forty undergraduate students at Rutgers University (Newark campus) provided similarity judgments between 1–7 for all possible 190 pairs [(20 × 19)/2] of faces generated by each prototype. Five participants were excluded for consistently responding with either 1 or 7, resulting in 16 subjects' similarity judgments for Prototype 1 and 19 subjects' similarity judgments for Prototype 2. Based on these similarity judgments we performed non-metric multidimensional scaling, producing a five dimensional psychological space, accounting for more than 90% of the original variance in the similarity judgments.

**Figure 2:**
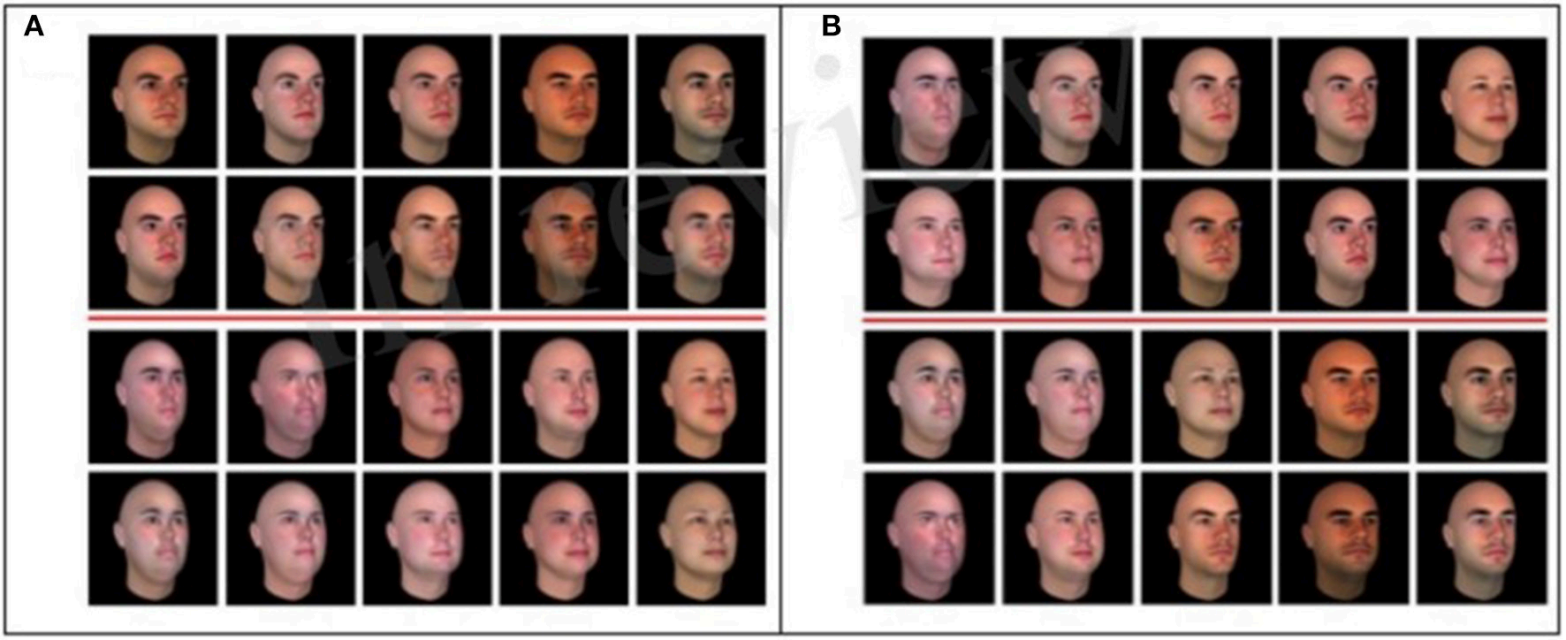
The final high dimensional **(A)** filtration separable (FS) and **(B)** condensation integral (CI) sets with the categorization lines indicated in red.

The five coordinates of the face stimuli in the derived psychological space were then used to create separable and integral stimulus sets based on low and high perceptual space distances respectively, by correlating (Pearson r) coordinates within each stimulus set. Faces for the integral category set were sampled from both prototypes, effectively increasing the dimensionality of the final integral category set, which at the same time had a relatively lower within category variance (r-within = 0.65) than between category variance (r-between = 0.42). This procedure creates a condensation category “rule” at the same time (see Figure 2A) since the shared dimensionality of the categories increases. Separable stimulus sets were constructed by sampling the same sets of ten faces from each separate prototype, thus decreasing the dimensionality within each category (r-within = 0.89) while increasing the perceptual distance between the two categories (r-between = 0.32) and creating a filtration rule on a low dimensional, highly correlated set per category (Figure 2B). For each category set, we used the exact same 20 stimuli from both prototypes selected to be used in the integral and separable stimulus sets. In effect, both the separable and integral category sets had the identical sample of 20 faces that were sorted into categories that in effect, induce separable and integral sets simply by manipulating the degree of correlation between the faces within and between each category.

### Tasks

Learning occurred under one of two conditions based on the category rule; one encouraging attention toward a small subset of relevant features (filtration task) and the other requiring the attention to, and integration of, a large number of the available stimulus features (condensation task). These tasks were drawn from those used in the Kruschke (1993) study in which attention was differentially oriented toward features.

### Subjects and procedure

Independent of the 40 subjects used for similarity scaling another 55 undergraduate students at Rutgers University in Newark participated in the experiments for course credit. We recruited for the low-dimensional task 20 subjects, while for the high dimensional task, another 31 subjects. In both tasks all participants were seated at a computer in a quiet room and first completed a warm-up task. The known categories of cats and dogs were used to familiarize the participants with the category learning procedure. Participants were told that they would be shown a series of eight pictures for 2 s each and would have to categorize the pictures by pressing either the right or the left arrow key. They were also told that in the beginning of the task they would not know what the categories were and on which arrow key they would be located on, but that the feedback after each trial would facilitate their learning. This procedure was the same for both Lo and Hi dimensional stimuli.

For the Hi-dimensional stimuli condition subjects were randomly assigned to either the FS (*N* = 16) or the CI condition (*N* = 15) using the high dimensional naturalistic stimuli. Before starting the experimental task, participants were given up to 4 min to study the distribution of all faces. The faces were arranged in a randomized order and participants could move on to start the experimental task by pressing any key. For the experimental filtration-condensation task participants saw 10 randomized repetitions of the 20 faces (2 s per face; 200 trials), which they had to categorize into the unknown categories of category A and category B by pressing the right and left arrow keys. Feedback about their answers was provided after each trial and at the end of the experiment a screen appeared informing the participant of their percentage of accurate answers. All participants but 2 of the subjects finished the lo-dimensional experiments with over 75% accuracy and those were excluded leaving 11 in the FS group while 9 in the CI group. Four participants were excluded from the hi-dimensional task due to insufficient learning (below 50% cumulative accuracy). The same exact procedures were used with the Lo-dimensional stimuli.

### Neural network modeling

To model the same filtration-condensation task as described for the human behavioral data, we used a simple fully connected feed-forward network with a single hidden layer (BP), as well as a fully connected feed-forward deep learning network with three hidden layers (DL). The goal was to observe the differences in learning dynamics and representational properties of the two networks with differing depths in architecture. Therefore, the BP and DL networks were kept as simple and similar to each other as possible, while only manipulating the depth of the architecture by increasing the number of hidden layers and also incrementally increasing the bottleneck with increasing layer to encourage feature extraction. Both networks used sigmoidal activation functions and the backpropagation learning algorithm. All modeling was done in R using the MXNet platform.

#### Low dimensional stimuli

Kruschke (1993) chose 8 symmetric exemplars that varied in rectangle height and line position within the particular rectangle (see Figure 1), The stimuli were represented using two sets for four binary-valued units; one set for the four possible line positions and one set for the four possible rectangle heights. One additional pair of binary-valued units was used for the category label^1^.

All stimuli were randomized and presented over 50 trials to the BP and 2000 (to equate the learning error equally asymptotically) to the DL networks, using initial randomization with weights randomly initialized between 1 and −1 with mean zero. Weights were updated either in 10 or 100 (this was constant for DL or BP within each case) epoch batches for stability. Results of a grid search confirmed the best number of hidden units for the BP network to be > 10 however, in order replicate Kruschke's original result we used the same number of hidden units (BP = 8-8-2) and for the DL network used the same number of 1st layer hidden units and added a small 2nd layer (DL = 8-8-3-2). Both networks performed the filtration-condensation task with the same learning parameters (learning rate = 0.1; momentum = 0.01, no dropout), using a sigmoidal activation function on all layers but the output layer, which used softmax in order to make the decision more similar to the human category decision (rather than forcing 100% certainty).

#### High dimensional stimuli

The same face stimuli used to collect the human behavioral data were down sampled, turned into feature vectors, and used as input (50 × 50 pixels, gray-scaled with values representing original color shading) for input to the BP and DL networks described above. Stimuli were randomized and presented to both types of networks with binary pairs as target values indicating category membership.

Networks were initially randomized with weights and biases, uniformly distributed between −1 and 1 with mean zero. The number of hidden units were selected in a grid search to maximize the fit to the accuracy data for the BP network (2500-250-2) and the DL network (2500-100-25-9-2). Again, both networks performed the filtration and condensation tasks with the same learning parameters for FS and CI (learning rate = 0.4; momentum = 0.01; dropout = 0.0002) these values were chose with a series of experiments, moderate learning rate was important for rapid learning even with small smoothing due to momentum and almost no dropout (however setting dropout to zero produced erratic results; this may have to do with the difficulty of the hi-D classification task compared to the lo-dimensional task), and the network had a sigmoidal activation function on all layers but the output layer, which used softmax.

## Results

### Behavioral results

#### Low dimensional stimuli

**Figure 3:**
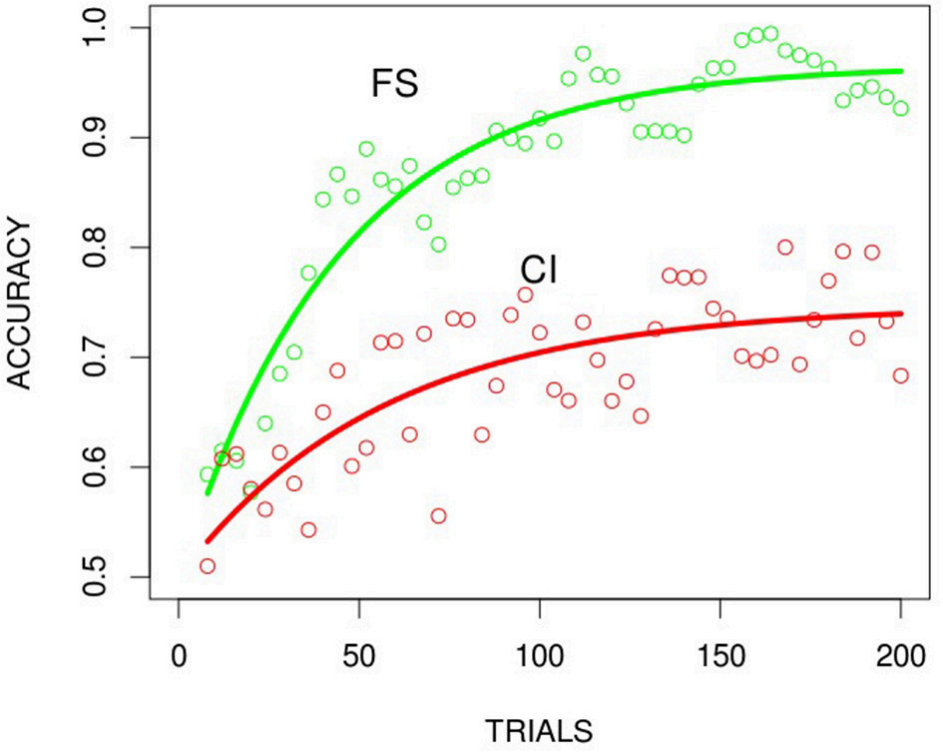
Human behavioral results for the 2D binary Kruschke (1993) stimuli for the FS and the CI conditions. Data fit with Equation 2: neg-exp (red FS and green CI). Data are binned (every 4 trials) and plotted at midpoints of each bin.

Learning curves for participants' accuracy over trials was generated for each condition to facilitate direct comparison of the speed and asymptote of learning across the FS (filtration-separable) and CI (condensation-integral) conditions. Cumulative accuracies were computed over all participants in the FS and CI conditions for the 200 trials and average binned in time every four trials creating 50 averaged plotted trials. Figure 3 shows that the behavioral results for humans learning to classify the Kruschke stimuli in the FS and the CI condition replicates the filtration-condensation phenomenon and the Kruschke (1993) results. Although participants initially have to hypothesize (subjects were debriefed after the session and asked about how they solved the task and what they thought the categories were) about which features are relevant, the filtration rule uses a single feature and can therefore be learned relatively quickly. In contrast, the learning curve of the CI task achieves asymptote at a slower rate, based on participants having to do more hypothesis testing to identify the stimuli with correlated features needed to “condense” or abstract the category rule (diagonal set in Figure 1A). We describe the learning curves in more detail in the curve fitting sections.

#### High dimensional stimuli

**Figure 4:**
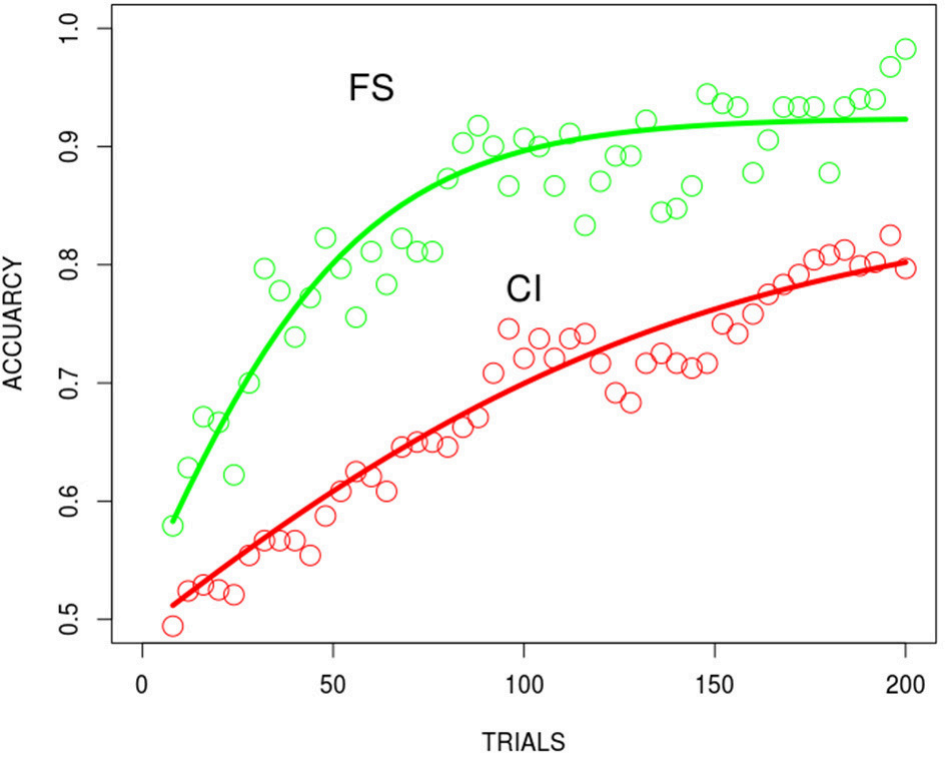
The human behavioral result for the FS and CI conditions using the high dimensional naturalistic stimuli. Best fitting functions, hyperbolic exponential functions, (red FS, green CI). Data are binned (every 4 trials) and plotted at the midpoints of each bin.

Learning curves for participants' accuracy over trials in the FS and CI conditions were generated in the same way as described for the low dimensional stimuli. Replicating the performance with low dimensional stimuli, the FS condition produced faster learning than the CI condition (see Figure 4). These results confirm that the prototypes of our naturalistic stimuli can be differentiated successfully and that selective attention is a source of the differential speed of learning. The curves in the plots are discussed in the following modeling section.

### Modeling results

#### Low dimensional stimuli

**Figure 5:**
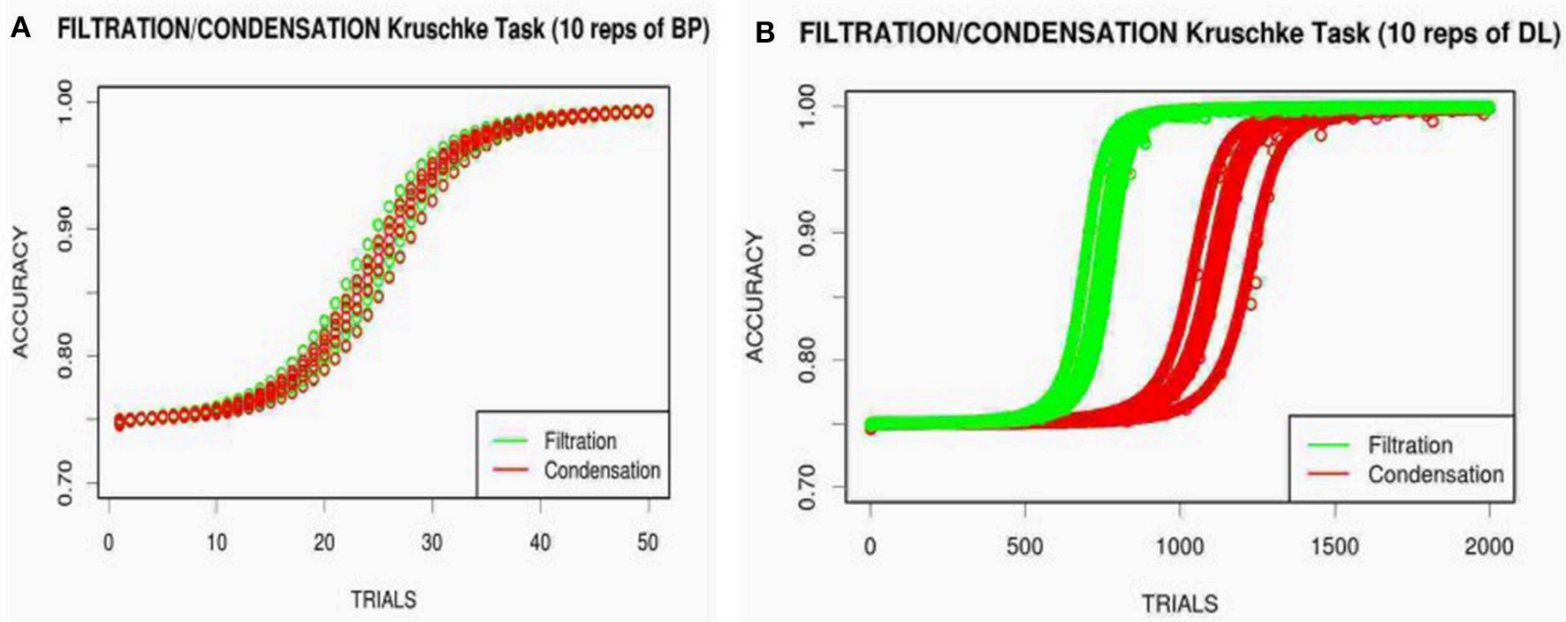
Modeling of the FS (green) and CI (red) conditions with the low dimensional Kruschke (1993) stimuli for BP **(A)** and DL **(B)** over 10 repetitions.

Displaying the average of 10 replications for each network, Figure 5 presents the learning curves of the BP and DL networks learning to categorize the Kruschke (1993) stimuli. Figure 5A shows that the BP network once again learned the FS and CI conditions at the same rate, replicating the failure of BP to show attentional modulation. The DL network however does show the differential learning speed with the FS condition being faster than the CI condition in Figure 5B. Note that, likely due to the stimulus encoding, the actual human learning is quite different from the neural network modeling. It would be possible to fine tune each network model independently to bring them into closer correspondence, but that would require different number of hidden units and parameter values. The DL network in particular was hampered by using small number of hidden units, as other experiments showed much faster learning with DL initialized with larger number of hidden units.

#### High dimensional stimuli

**Figure 6:**
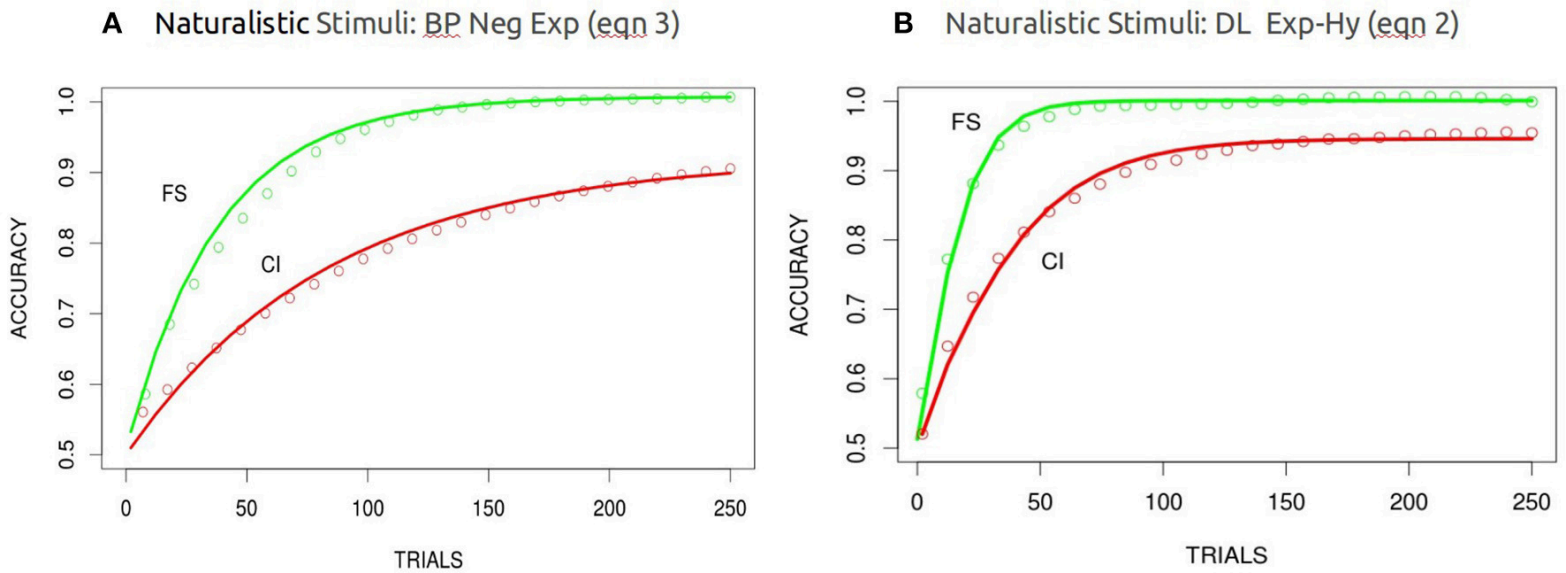
Modeling of the FS and CI conditions with the high dimensional naturalistic stimuli for BP **(A)** and DL **(B)** averaged over 100 runs. Best fitting functions were the negative exponential functions for BP and the hyperbolic exponential functions for DL (overlaid in red). These are binned averages over blocks of 100, there were a total of 250 weight updates.

The learning curves (averaged over 100 independent runs and batched at 10 trials each—consequently there were 250 learning trials per network) for the BP and DL networks in the FS and CI conditions using the high dimensional stimuli are shown in Figure 6. The DL network exhibits the differential learning speed between the filtration and condensation conditions as it had using low dimensional stimuli. However, in contrast to the performance of the BP network when processing low dimensional stimuli, the BP network now successfully replicates human learning performance when given the analog high dimensional stimuli. Thus, consistent with our second hypothesis, it appears that increasing the complexity of category structure beyond simple binary feature values slows learning by the BP network in the CI condition and makes its learning rate consistent with that of humans and the DL network.

#### Fit to learning curves

In order to identify potential functions that might fit the accuracy over trials we compared the learning curves for BP and DL simulations across the various stimulus sets and conditions. From a qualitative point of view there is a salient difference in the learning curves with the low-D stimuli for both models in that they exhibit a long latent period (performance near chance) before beginning to rise, unlike the learning curves for the human participants with either type of stimuli and unlike both models with the Hi-D natural stimuli. In all of these cases, accuracy starts to rise above chance from the very first trial, and the slopes of the learning curves decrease monotonically in all of these cases. Because this is more of a curve fitting exercise we are considering number of different common functions used for learning curves. To examine the differences in shape and asymptote, we used two common learning curve functions (Mazur and Hastie, 1978) to determine the best fitting function, accounting for the highest percentage of variance as well as a more general Gompertz type model (where T = trials, a = shape factor, and b = scale factor) which is known to fit a variety of shapes^2^.

1. Accuracy = 1/(1+exp(b^*^(1−exp(−a^*^T)))) “gompertz”
2. Accuracy = b^*^(exp(−0.5−a^*^T)/(exp(−0.5−a^*^T)+1)) “exponential-hyperbolic or logistic”
3. Accuracy = b^*^(1−exp(−0.5−a^*^T)) “negative exponential”

Again this is not an attempt to construct a mechanistic or predictive model, but rather a systematic characterization of shape and form. In the case of BP (simulations both FS and CI conditions) learning curves, a near perfect fit was found with a (3) negative exponential function and for DL (simulations mainly with FS conditions) with an (2) exponential-hyperbolic function (logistic) (Figure 6) for the Hi-dimensional or “naturalistic” stimuli. Both functions accounted for more than 99% of the variance (see Table 1). Equation 1, the Gompertz was similar in fit to the negative exponential and accounted for less variance (average 89%) for the DL simulation learning curve. The different functions, characteristic of BP and DL learning dynamics underlying DL learning patterns, may reflect the different dynamics of each algorithm (see Saxe et al., 2014). Fitting these same functions (intercept was fixed to 0.5 where required, for the classification task and the asymptote was allowed to vary as a function of a scale and shape parameter see Table 1 below) to the human behavioral data for the Lo-dimensional FS task (Figure 3), and FS was best fit with the negative-exponential (Equation 3) while the CI task showed to be indeterminate and fit equally well with the negative-exponential and the exp-hyperbolic (Equation 2). The fit to the Hi-dimensional FS task (Figure 4) also favored the exponential-hyperbolic function (Equation 2) accounted for 93% of the variance, while the negative-exponential function (Equation 3) only accounted for 84% of the variance also confirmed by log-likelihoods (where difference in R^∧^2 was insignificant, Log-likelihoods-with higher sensitivity- were compared). The Gompertz model (Equation 1) provided adequate fits to the Lo-dimensional data compared to the negative exponential, and similarly to the Hi-dimensional data (90% in hi-dimensional) but never exceeded the fits of the other two models.

**Table 1:** Model fits to subject and simulation learning.

**Fits to data**		**Human data**				**Model Hi-D**		**Model Hi-D**	
**Data type**		**Lo-D**		**Hi-D**		**BP simulation**		**DL simulation**	
**Model**	**Cond**.	**FS**	**CI**	**FS**	**CI**	**FS**	**CI**	**FS**	**CI**
**Neg exp**	R^∧^2	96^*^	90	84	98	**99**	**99**	99.2	99.7
	LL	**95.9**	89.5	72.7	120	77.7^*^	85.5^*^	67.0	88
Param scale,		0.02	0.010.24	0.05	0.005	0.04	0.02	0.06	0.04
shape		0.46		0.61	0.48	0.99	0.42	0.50	0.44
**Hyp-exp** (logistic)	R^∧^2	92	89	93^**^	98	98.7	92.0	**99.8**	99.3
	LL	89.0	83.9	**97.7**	121	69.3	65.6	70.7^*^	85
Param scale,		0.02	0.03	0.03	0.01	0.04	0.02	0.08	0.04
shape		1.04	1.35	1.08	1.16	1.00	1.09	1.00	1.05
**Gompertz**	R^∧^2	86	87	89	94	97	97	98	94
	LL	74.5	76.5	82.4	96.1	58.8	50.3	67.6	39.8
Param scale,		0.01	0.005	0.007	0.008	0.02	0.006	0.05	0.01
shape		0.62	0.42	0.84	0.29	0.52	0.58	0.49	0.68

* *0.1*,

** *0.05. Bold entries indicates a statistically significant value above all others in that column*.

#### Hidden unit representation

We conducted an analysis of the internal representations of the hidden units at all layers of the network. These hidden unit representations at the weight layer illustrate how BP and DL differ in processing category exemplars.

**Figure 7:**
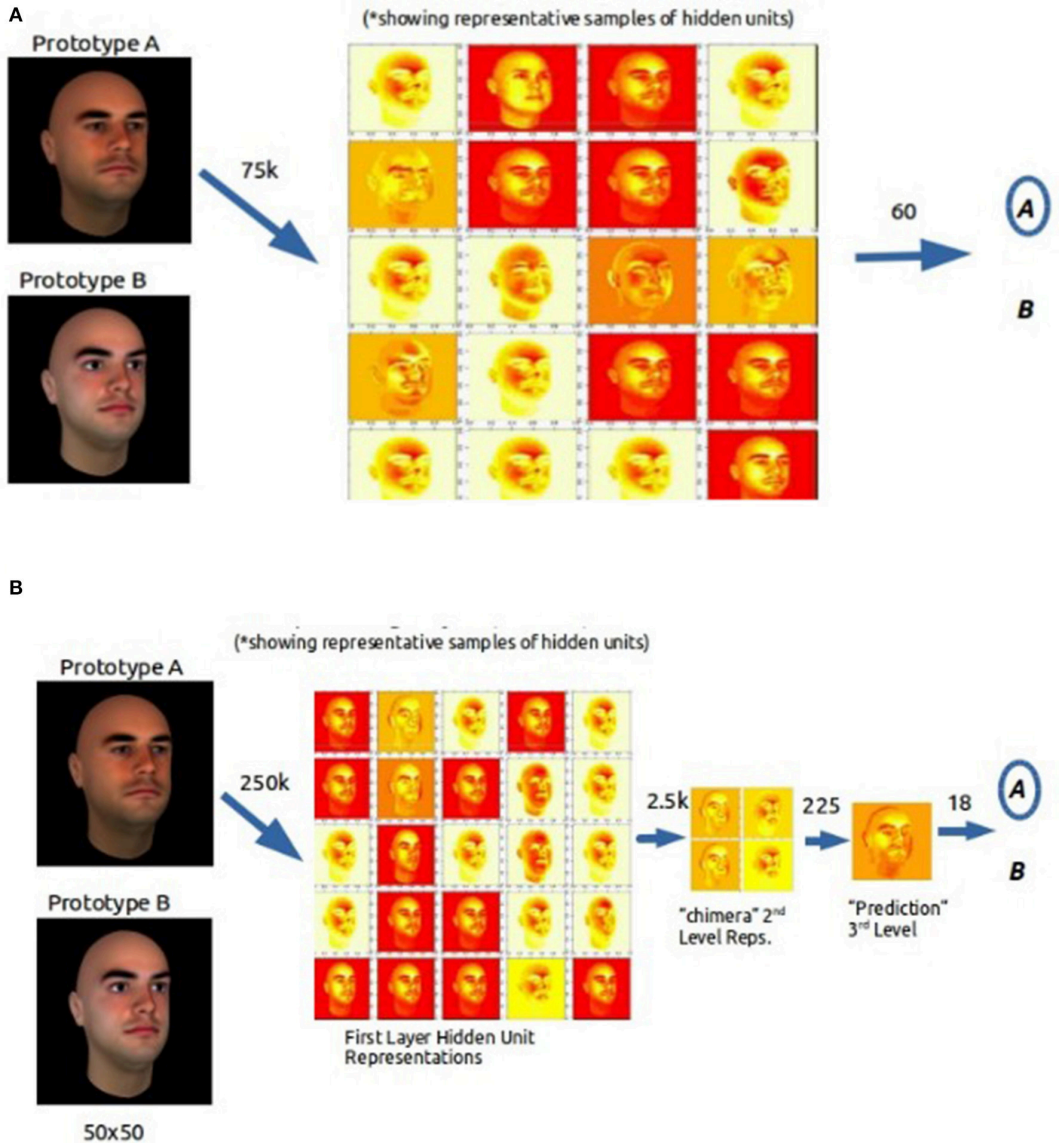
Visualizations of the internal representations of a representative sample of the hidden units in the BP **(A)** and the DL **(B)** learning categories in the FS condition.

Once learning was complete, we plotted the weights at each layer of the network with a heat map (see Figure 7), where lighter colors represent increasingly stronger weights (white = strongest). The weights of the hidden units in the BP network show a diversity of values for face structures (e.g., eyes, mouth, forehead) which are then used to predict category membership in the subsequent output layer.

Looking very similar to the first layer of the BP network, the first layer of the DL network shows a diversity of weightings on face structures. However, in successive layers, DL appears to use a progressively finer decision structure that ultimately produces a weighted prototype of the two categories. For example, the second layer uses first layer output to construct more complex abstractions, or feature hybridization, based on re-weighting features from first layer. At the third layer, a specific category prototype (that was not trained on) appears that allows a final filtering of the input and a decision on the category value of the input.

## Discussion

The complexity of category structure, and attentional bias toward stimulus features are well known factors in category learning performance. An early demonstration (Shepard et al., 1961) used six types of categories in which the feature combination defining each category followed various boolean rules. These category rules varied from simple conjunction (linearly separable) to complex disjunctions (non-linearly separable). Learning rate decreased dramatically as category rule complexity increased. Analysis of subject errors and sequential performance indicated that when rule complexity demanded attentional binding over two or more features, learning rate decreased. The Garner/Kruschke condensation/filtration task is in some ways a variant of the Shepard et al. (1961) study.

In general, then, categories having a separable feature structure are learned much more easily than those having an integral feature structure. This effect of category structure is enhanced when attention is appropriately directed toward stimulus features. Specifically, attention toward a subset of features relevant to the category rule increases the rate of learning for categories with a separable structure, as does attention toward the integration of available features in the case where the category structure is integral. What has not been known is why single hidden layer BP networks, unlike humans or DL networks, are able to learn categories with an integral structure as easily as those with a separable structure. We believe we can provide a reasonable account of why the learning performance of unmodified BP networks is inconsistent with that of humans and DL networks, and moreover, why DL networks are so successful at learning across a wide and diverse range of categorization tasks.

We approached the problem of why the unmodified BP network seemed unable to modulate attention toward stimulus features by examining the roles played by category structure and attentional bias during category learning. We explored two possible hypotheses. One hypothesis focused on the limitations of the BP network architecture to differentiate separable and integral category structures. Specifically, we believed that the unmodified single hidden layer BP network cannot accommodate the feature binding and feature competition (Hanson and Gluck, 1991) that are necessary to respond differentially to differences in category structure complexity. A second hypothesis focused on the sensitivity of the unmodified BP network to the analog nature of the stimulus structure. Prior research examining category learning with BP networks used stimuli with feature dimensions that were binarized, rather than continuous. So, for example, the Kruschke (1993) study used stimuli having four rectangle lengths and four segment positions, although these two dimensions are inherently continuous. We hypothesized that this restricted use of feature dimensions artificially limited stimulus complexity which in turn limited the attentional bias of the BP network.

Using the original 2D binary Kruschke (1993) stimuli, we showed that the DL network, as opposed to the BP network, do in fact learn the filtration-condensation tasks at differential speeds, producing the human-like attentional bias found in category learning. These findings confirm our hypothesis that the DL network, like human subjects, can find structure in the same task at which the BP network failed 30 years ago (despite the binary encoding). This result provides the first computational model (DL) to successfully replicate qualitative learning speed of the diagnostic filtration-condensation performance of human category learning, without requiring hand-engineered or pre-wired adaptive mechanisms.

It is important to note a number of failings of the existing modeling approach. First in the lo-dimensional task due to attempting to equate the learning parameters and network sizes, making them similar to the Kruschke (1993) networks, DL was hampered by this constraint and considerably slower at learning than humans with the same task. It is also interesting to speculate whether the analog nature of the Kruschke stimuli might have made any difference. It seems unlikely as the number of bits inherent in the analog encoding is no more than the 8 bit encoding we used for the same information and that incrementally extracting would be faster. The human data, especially in the condensation task was difficult for subjects and produced slow and highly variable learning curves which made curve fitting indeterminate, even though the learning rates between FS and CI were different. In the hi-dimensional task, both the BP and DL models were consistent with the learning time of the human subjects. This is likely due to the higher complexity of the pixel input rather than just the analog nature of face stimuli. One possible hypothesis for this effect, involves the concomitant increase in the size of the network architecture with Hi-Dimensional stimuli. Similarly to a DL network, simply having more weight connections may allow for a segregation of network weights and hidden units into those with stronger gradients and those with weaker ones allowing for a faster rate especially in the FS stimuli. In effect, the increase in architecture size whether due to extra layers or Hi-Dimensional input would allow in differentiation between the FS and CI conditions.

We also showed that faces, which are high dimensional stimuli, can be used to construct separable and integral stimulus sets by simultaneously varying the within-category and between-category correlation of stimulus features. Human performance with these stimuli replicated that in which categories based on binarized stimulus features had been used. Specifically, learning was faster for the face category in which a filtration rule was used with separable features (FS condition) than that in which a condensation rule was used with integral features (CI condition). Moreover, we found that the BP network, like humans and the DL network, can successfully model human category learning when high dimensional stimuli are used. Thus, as we hypothesized, the unmodified BP network can, in fact, learn attentional feature binding. However, unlike humans and DL, the BP network needs the analog stimulus constraints present in the naturalistic stimuli to reproduce the human like attentional bias. To our knowledge, this is the first case of a simple single hidden layer BP neural network to model human attentional bias in category learning without engineering the network attentional bias.

Whereas the BP and DL networks both successfully modeled human performance when learning high dimensional stimuli (faces), the two networks did not apparently learn in the same way. An examination of the learning dynamics show qualitatively different learning curves for the BP network and the DL network. The learning of the BP network is best fit by a negative exponential function^3^ over trials, whereas the DL network is best fit by an exponential hyperbolic function.

A second goal of this study was to understand how the architecture of the DL network is able to accommodate the interaction of category structure complexity and attentional bias toward stimulus features.

We wondered what it was about the DL network architecture specifically that yielded such success in a wide diversity of categorization tasks. By comparing performance of the unmodified BP network with that of the DL network, and examining the hidden unit representations developed during learning, we hoped to obtain some understanding of why the DL network, but not BP network, learns categories in the way that humans do.

Since their conception, artificial neural networks have increased in the number of hidden layers they can accommodate. Simultaneously, this has also increased the complexity of problems they can solve. In the early days, the simple mapping of input to output of the perceptron (Rosenblatt, 1961) could only solve linearly separable problems. The addition of a single hidden layer between input and output in the BP neural network (Rumelhart and McClelland, 1986) improved the ability of the BP network to solve problems having complex category structure. However, although the addition of one hidden layer in the BP network substantially increased the network's ability to solve complex decision boundaries, the model itself still had difficulty creating representational structures. The advent of the DL network showed that even more complex decisions can be handled by adding more layers between input and output (Hinton et al., 2006). The additional layers of the DL network seem to aid complex categorization decisions by increasing abstraction of the representational structures from layer to layer.

An analysis of the internal representation of the hidden units indicates that the BP and the DL networks use distinctive processing strategies. While the representations at the first hidden layer are the same for the BP network and the DL network, the BP network must base its final category prediction solely on these first layer representations. These first layer representations are not conducive to feature competition. The DL network on the other hand uses additional, subsequent layers to abstract away from the raw feature input to create higher level representations of the category. We propose that this successive recoding leads to sequential extraction of features and the development of more sensitive feature detectors having higher fidelity and more attentional bias than is possible within a single hidden layer.

## Ethics statement

This study was carried out in accordance with the recommendations of Institutional Review Board in the Office of Research Regulatory Affairs with written informed consent from all subjects. All subjects gave written informed consent in accordance with the Declaration of Helsinki. The protocol was approved by the Institutional Review Board in the Office of Research Regulatory Affairs.

## Author contributions

LC, SH, and CH: Designed the study and analyzed the data; LC: Collected the data and drafted the article; SH and CH: Wrote final version.

### Conflict of interest statement

The authors declare that the research was conducted in the absence of any commercial or financial relationships that could be construed as a potential conflict of interest.

## Footnotes

^1^In the original Kruschke study the input encoding was based on the height of the rectangle and the position of the line within the rectangle. Although height and position are inherently continuous, the eight pairs of values were chosen using the same values per dimension, effectively making the feature values binary. We used an 8 bit binary (16 values) encoding which was potentially more expressive and nonetheless the results were identical to the original two dimensional encoding, which we also replicated.

^2^This form was suggested by J. McClelland.

^3^The exponential-hyperbolic (logistic) learning curve possesses a range of shapes that are distinctive compared to the slow gradual rate of change of the negative exponential (see Mazur and Hastie, 1978). It is worth noting that the exponential-hyperbolic can exhibit behavior from a sigmoidal shape (with a long dwell phase) or a extremely fast rise to almost an immediate asymptote. In the present case the low-dimensional binary stimuli in the Kruschke/Garner task was more sigmoidal and quite different from the human performance on the same task (both in shape and number of trials). This might be due to the fact that the task was presented in an analog form to human subjects and in a binary form to the BP networks, although as discussed above this seems unlikely. In the Hi-D case, the DL and the human performance were quite similar in both shape and number of trials.
